# Respectful maternity care during labor and childbirth and associated factors among women who gave birth at health institutions in the West Shewa zone, Oromia region, Central Ethiopia

**DOI:** 10.1186/s12884-020-03135-z

**Published:** 2020-08-03

**Authors:** Gizachew Abdissa Bulto, Dereje Bayissa Demissie, Abera Shibru Tulu

**Affiliations:** 1 Department of Midwifery, College of Medicine and Health Sciences, Ambo University, Ambo, Ethiopia; 2 Department of Neonatal Nursing, Saint Paul’s Hospital Millennium Medical College, Addis Ababa, Ethiopia; 3 Department of Public Health, College of Medicine and Health Sciences, Ambo University, Ambo, Ethiopia

**Keywords:** Respectful Maternity Care, Labor and Childbirth, Health Institutions, West Shewa Zone

## Abstract

**Background:**

Skilled assistance during pregnancy and childbirth is one of the key interventions in reducing maternal morbidity and mortality. But studies have shown that many women across the globe experience disrespectful and abusive treatment during labor and childbirth in institutions, which forms an important barrier to improving skilled care utilization and improving maternal health outcomes. Although there are few studies done in Ethiopia, information on the status of respectful maternity care (RMC) among women during childbirth at health institutions in the West-Shewa zone is lacking. Therefore, the study aimed to assess RMC during Labor and Childbirth and associated factors among women who gave-birth at health-institutions in the West Shewa zone, Central Ethiopia.

**Methods:**

Cross-sectional study was conducted at Health institutions in the West Shewa zone, Oromia region, Central Ethiopia. A systematic random sampling technique that uses women’s delivery registration number was used to collect data. Data was collected through an exit-interview. Both bivariate and multivariable logistic regressions were used to identify associated factors.

**Results:**

From a total of 567 women who fully responded, only 35.8% received RMC. From categories of RMC, 76.5% of the woman is protected from physical harm/ill-treatment and 89.2% received equitable care free of discrimination. But, only 39.3% of woman’s right to information, informed consent and preferences were protected. Giving birth at health center (AOR:5.44), discussion on the place of delivery (AOR:4.42), daytime delivery (AOR:5.56), longer duration of stay (≥ 13 h) (AOR:2.10), involvement in decision-making (AOR:8.24), asking for consent before the procedure(AOR:3.45), current pregnancy unintended (AOR:5.56), the presence of < 3 health-workers during childbirth (AOR:2.23) and satisfied on waiting-time to be seen (AOR:2.08) were found to be significantly associated with RMC.

**Conclusions:**

The proportion of RMC during labor and childbirth in the study area was low. Type of institution, discussion during ANC, time of delivery, duration of stay, involvement in decision-making, the number of health workers, waiting time and consent were identified factors. Therefore, giving emphasis to creating awareness of care providers on the standards and categories of RMC, improving care provider-client discussion, monitor and reinforcing accountability mechanisms for health workers to avoid mistreatments during labor and childbirth were recommended.

## Background

Respectful maternity care (RMC) is an approach centered on an individual, based on principles of ethics and respect for human rights, and promotes practices that recognize women’s preferences and women’s and newborns’ needs [1, 2]. RMC is a universal human right that is due to every childbearing woman in every health system [3, 4]. However, many women across the globe experience disrespectful, abusive or neglectful treatment during childbirth in health institutions [5]. The reported forms of Dis-Respect and Abuse (DRA) have been classified into seven categories: physical abuse, non-consented care, non-confidential care, non-dignified care, discrimination based on patient’s attributes, abandonment of care and detention in facilities [6].

Evidence from various countries in Sub-Saharan Africa shows that women would prefer to deliver in a facility, but choose not to because of previous experiences of inadequate, low quality, and/or disrespectful care in facilities[7–9]. Despite this, reports of “disrespectful and abusive treatment” during labor and childbirth continue to appear in the world [5, 7, 10]. Given the abundant reports of disrespectful and abusive obstetric care, women in low and middle-income countries fear various undesirable procedures and may prefer to deliver at home with a traditional birth attendant [4, 11].

Different studies highlighted the connection between disrespect and abusive care during facility-based childbirth and a decision by women not to use facility-based childbirth services. The negative effect of DRA during childbirth on skilled delivery attendance constitutes an important barrier to increasing services utilization and enhancing maternal well-being [8, 12, 13]. In Tanzania women who experienced disrespect/abuse were less likely to plan to deliver again at the same facility [14]. Bowser and Hill found that a weak health care system and shortages in human resource contributes to disrespectful or abusive care. Health facility infrastructure, resources and commodities were mainly mentioned as a contributors for the lack of RMC [15].

Skilled assistance during pregnancy and childbirth is one of the most important interventions in reducing maternal morbidity and mortality. Efforts to increase the use of facility-based maternity care in low-income countries are unlikely to achieve the desired gains if there is no improvement in the quality of care provided, especially elements of respectful care [16]. The negative patient experiences at health institutions contribute to poor health outcomes and reinforce mistrust of institutional care. Additionally, women and families may delay or avoid seeking care in health facilities, even at the risk of their own health and that of their newborn [17, 18].

Ethiopian federal ministry of health in its Health Sector Transformation Plan (HSTP) has planned to increase the level of deliveries attended by skilled birth attendants from 15% in 2014 to 90% by 2020. To help achieve the targets set, the Ministry has identified caring, respectful and compassionate (CRC) health professionals as one of the four transformation agendas. Lack of respect for patients and their families is a common complaint and having CRC health professionals is a critical requirement to ensure equity and achieve high-quality health services on its HSTP [19, 20].

In Ethiopia, the pooled prevalence of disrespect and abusive care during childbirth and maternity care was 49.4% [21]. More than two-thirds (78.6%) of postpartum mothers in Addis Ababa, 67.1% in Bahir Dar city and 21% of women in four health centers of Amhara and Southern regions of Ethiopia reported as they experienced one or more categories of disrespect and abuse during labor and childbirth [22–24]. Another study in Bahir Dar city showed only 57% of mothers received RMC [25].

As indicated, disrespect and abuse during labor and childbirth is an important concern, especially in countries like Ethiopia, where the maternal mortality rate is high (412/100,000 live births) and yet the skilled birth attendance has been very low (28%) [26].

In Ethiopia, even if there are few studies conducted on the status of disrespect and abusive treatments, data on the status of respectful maternity care and associated factors among women who give birth at health institutions in the west Shewa zone is lacking. Therefore, this study aimed to assess RMC during Labor and Childbirth and associated factors among “women who gave-birth” at health-institutions in West Shewa zone, Central, Ethiopia. Findings from this study may also help policymakers, program managers and organizations working on this area as baseline data for effective implementation of CRC in the HSTP, improving DRA treatments at health institutions.

## Methods

### Study design, period and area

An institution-based cross-sectional study design was used to assess the status of RMC during Labor and Childbirth and associated factors among women who gave birth in public Health institutions of West Shewa zone, Central Ethiopia, from April 01 to June 30, 2018. Ambo town which is the capital of the zone is located 114 km to the west of Addis Ababa, the capital of the country. Available information from the zonal health office shows that the total population of the zone is estimated to be 2,381,079 of which 1,214,350 is female. Currently, the health system of the zone consists of one university referral hospital, one general Hospital, five primary hospitals, ninety-two health centers and four hundred forty-seven health posts with 98% of potential health service coverage. All health centers and hospitals provide 24 h of labor and delivery services [27].

### Source population and study population

The source population was all women who delivered at health institutions in the West Shewa zone. The study population was all women who delivered at the selected Health institutions during the data collection period and selected by a systematic random sampling. Women who were referred from other health institutions after giving birth to those selected health institutions were excluded.

### Sample size and Sampling procedure

The sample size was determined by using single population proportion formula with the assumption that 78% proportion (P) of women experienced one or more categories of disrespect and abuse from a study done at health facilities in Addis Ababa [22], at 5% level of significance and a margin of error of 5%. By considering a 10% non-response rate and design effects of 2, the final sample size was 582.

A simple random sampling technique was utilized to select 3 hospitals and 19 health centers that have more delivery. The total sample sizes were allocated proportionally to each of the selected hospitals and health centers by reviewing the number of deliveries attended to by each health facility. A systematic random sampling technique was used to collect data using women’s delivery registration number from the delivery logbook. Data were collected from every 3rd woman who gave birth during the study period at each selected health institutions.

### Operational definitions

In this study, women were considered to have received respectful maternity care during labor and childbirth if they answered yes to all of those questions assessing RMC or verification criteria used for assessing the seven categories (performance standards) of RMC during labor and childbirth [22, 25, 28].

Women were considered as experienced disrespect and abuse if they answered no to one or more of those questions assessing RMC or verification criteria used for assessing the seven categories of RMC [10, 12, 23].

### Data collection tools and procedures

The questionnaires for data collection were initially prepared in English, and translated into the local language (Afan Oromo) and back into English to check for consistency with language experts. Data was collected through an exit interview by using a pre-tested structured Afan Oromo version questionnaire. We used a validated tool for assessing RMC which was adapted from the Maternal and Child Health Integrated Program. The other included questions in the questionnaire were prepared by reviewing different other related works of literature and variables identified to be measured [25, 28, 29].

Twenty-four [24] data collectors who were not working in the study area were recruited for the data collection and three [3] Master Degree holders conducted supervision during the data collection period. The training was given for data collectors and supervisors by investigators for two days. Pre-test of the questionnaire was done on 5% of the women who delivered at Holeta health center and Inchini hospital, to identify any ambiguity, check for consistency of the questionnaire, acceptability and necessary correction were made one week before the actual data collection. The filled questionnaires were collected and checked for consistencies and completeness daily by supervisors and principal investigators.

### Data processing and analysis

The returned questionnaires were checked for completeness, cleaned manually, coded and entered into EPI Data version 3.1software and then exported to SPSS windows version 23 for further analysis. Bivariate analysis was used primarily to check which variables have an association with the dependent variable individually. Variables which were found to have an association with the dependent variable (*p*-value ≤ 0.2) were then entered into Multiple Logistic regression for controlling the possible effect of confounders and finally the variables which have significant association were identified based on AOR, with 95%CI and *p*-value ≤ 0.05 to fit into the final regression model.

### Ethical considerations

Ethical clearance was obtained from the research review and ethics committee of Ambo University, collage of Medicine and Health Sciences.

## Results

A total of 567 women fully responded to the interview questionnaire making a response rate of 97.4%. The majority of respondents, 263 (46.4%) had their childbirth in health centers, 546 (96.3%) were married, 303 (53.4%) were protestant religion followers, and 530 (93.5%) belongs to the Oromo ethnic group. Regarding women’s occupational status 202 (35.6%), were housewives and about 395 (69.7%) were urban residents. One-fourth of the respondents attended primary education 150 (26.5%). The mean age of respondents was 26.9 years with a standard deviation of 5.2 years (Table 1).

**Table 1:** A Socio-Demographic characteristic of mother’s who gave birth at public health facilities in the west Shewa zone, Oromia region, central Ethiopia, 2018

Characteristics	Categories	Number (*N* = 567)	Percent (%)
Type of Institution	Health center	263	46.4
	Primary Hospital	157	27.7
	General Hospital	147	25.9
Age in years	17–24	192	33.9
	25–29	205	36.2
	More than 29	170	30.0
Marital Status	Married	546	96.3
	Other marital Status^a^	21	3.7
Religion	Orthodox	225	39.7
	Protestant	303	53.4
	Muslim	31	5.5
	Other Religion^b^	8	1.4
Ethnicity	Oromo	530	93.5
	Amhara	30	5.3
	Other Ethnicity^c^	7	1.3
Educational Status	Unable to read & write	86	15.2
	Able to read and write	60	10.6
	Primary education	150	26.5
	Secondary Education	133	23.5
	Collage and above	138	24.3
Mothers Occupation	Government Employee	123	21.7
	Housewife	202	35.6
	Farmers	85	15.0
	Merchant	78	13.8
	Private Employee’	66	11.6
	Other Occupation^d^	13	2.3
Mothers Residence	Urban	395	69.7
	Rural	172	30.3
Average Monthly Income	Less than 2000	294	51.9
	More than 2000	273	48.1
	Mean = 2787.1 with SD of 2450.85 Ethiopian Birr, Median = 2000.00		

Keys: ^a^Divorced, widowed & single, ^b^Wakefata & Catholic, ^c^Gurage & Tigre, ^d^Student & Daily laborers

### Obstetric Related Characteristics of Women

The result of this study indicated that the majority of respondents 417 (73.5%) were multiparous and 537 (94.7%) had ANC follow up. Of the total respondents, 453 (79.9%) of them gave birth with spontaneous vaginal delivery, 549 (96.8%) were live birth, and in only 54 (9.5%) of them, there were cultural practices (coffee or porridge) after delivery (Table 2).

**Table 2:** Obstetric characteristics of mother’s who gave birth at public health facilities in the west Shewa zone, Oromia region, central Ethiopia, 2018. (*N* = 567)

Characteristics	Categories	Number	Percent (%)
Number of Parity	Primi-para	150	26.5
	2–3	211	37.2
	≥ 4	206	36.3
Current pregnancy Intended/wanted	Yes	507	89.4
	No	60	10.6
Number of ANC follow ups	No ANC follow up	30	5.3
	Once to twice	90	15.9
	Three times	176	31.0
	Four and above	271	47.8
Providers discussed about place of delivery during ANC	Yes	462	81.5
	No	75	13.2
	No ANC follow up	30	5.3
Discussed place of delivery with partner	Yes	409	72.1
	No	158	27.9
Visit type for current delivery	New or My first time	225	39.7
	Repeat Visits	298	52.6
	Referred from other institution	44	7.8
Labor started	Spontaneous	526	92.8
	Induced	41	7.2
Duration spent on labor	Less than 12 h	416	73.4
	More than 12 h	151	26.6
Total current stay at Health facilities	12 h or less	277	48.9
	13 to 24 h	147	25.9
	25 h and above	143	25.2
Mode of Delivery	Spontaneous vaginal delivery	453	79.9
	Assisted vaginal Delivery	45	7.9
	Cesarean section	69	12.2
Outcomes of delivery	Alive	549	96.8
	Stillbirth	18	3.2
Condition of mother during childbirth	No complication	498	87.8
	Had Complications	69	12.2
Time of delivery	Day time	301	53.1
	Nighttime	266	46.9
Who attended your delivery	Midwife	420	74.1
	Nurse or Health Officer	39	6.9
	Doctors or Emergency Surgeon	108	19.0
Sex of Provider	Male	287	50.6
	Female	280	49.4
Procedures Done	Episiotomy	142	25.0
	Fundal pressure	65	11.5
	Manual removal of placenta	24	4.2
	Instrumental Delivery	39	6.9
	Cesarean Section	69	12.2
Asked your consent before performing procedure	Not asked me	79	13.9
	Yes	240	42.3
	No Procedure done for me	248	43.7
Number of attendants during childbirth	One	73	12.9
	Two	240	42.3
	Three	111	19.6
	Four and above	143	25.2
Anyone other than concerned provider gets access to see you during LAD	Yes	92	16.2
	No	475	83.8
There are cultural practices for mothers after delivery (Coffee or porridge)	Yes	54	9.5
	No	513	90.5
Admitted to maternity waiting home before labor started	Yes	32	5.6
	No	535	94.4
Do you want to have a child in the future	Yes	433	76.4
	No	134	23.6

### Prevalence and Categories of Respectful Maternity Care

The overall proportion of women who received RMC during labor and childbirth was 203 (35.8%) [95% CI: 31.7–39.7], but a significant number of women 364 (64.2%) had experienced disrespect and abusive care during childbirth [95% CI: 60.3–68.3]. Only 47.3% of women who gave birth at health centers and 25.8% who gave birth at hospitals received RMC ($x2=27.8$, *P*=0.000).

**Fig. 1:**
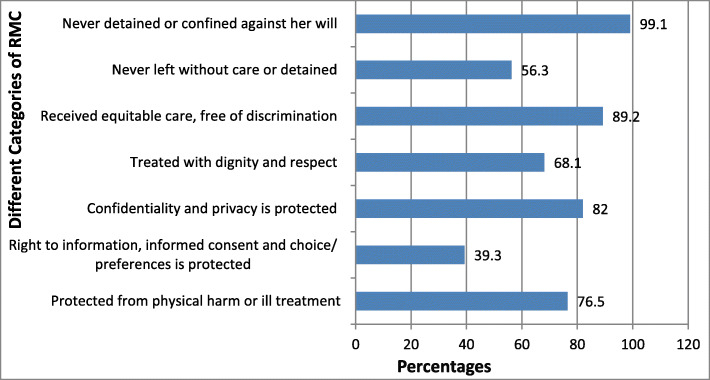
Prevalence of different categories of RMC during childbirth at public health facilities in West Shewa zone, Oromia region, Ethiopia, 2018

From categories of RMC, 434 (76.5%) of the women were protected from physical harm or ill-treatment and 506 (89.2%) received equitable care free of discrimination. More than half 319 (56.3%) of respondents were never left without care or unattended. But, only 223 (39.3%) of a woman’s right to information, informed consent, and preferences were protected (Fig. 1).

From the categories of being free from physical harm or ill-treatment, 498 (87.8%) of mothers were never physically forced or abrasively handled by care providers and 416 (73.4%) of them were provided comfort or pain relief as necessary.

Woman’s right to information, informed consent and choice or preferences is the least respected from categories of RMC. In about 419 (73.9%) of women, they were allowed to move during labor and 312(55.0%) were allowed to assume a position of choice during birth. For about 369 (65.1%) of women care providers responded to their questions with promptness, politeness and truthfulness and 356 (62.8%) of them explained what is being done and to expect throughout childbirth.

Regarding the confidentiality and privacy of the respondents, the providers kept a patient’s file in limited access areas for 531 (93.7%) of them and used drapes or covering to protect their privacy in 487 (85.9%). Four hundred and forty-two (78.0%) of women were not insulted, intimidated, threatened, shouted at, scolded, laughed, scorned or coerced. In about 519 (91.5%) of respondents, the provider did not show disrespect to the women based on some attributes. The women were never left without care or unattended in 353 (62.3%) of them and 563 (99.3%) were not forced to stay against their will (Table 3).

**Table 3:** Proportions of different categories of RMC during labor and Childbirth at public health facilities in West Shewa Zone, Oromia region, Ethiopia, 2018. (*N* = 567)

Categories of RMC	RMC		DRA	
	Number	Percent	Number	Percent
1. **The woman is protected from physical harm or ill-treatment**				
Never used physical force/abrasive behavior with the woman	498	87.8	69	12.2
Never physically restrains woman	504	88.9	63	11.1
Touches/demonstrate caring in a culturally appropriate way	530	93.5	37	6.5
Never separates woman from her baby unless	552	97.4	15	2.6
Does not deny food or fluid to women in labor	550	97.0	17	3.0
Provides comfort/pain-relief as necessary	493	86.9	74	13.1
2. The woman’s right to information, informed consent and choice/ preferences is protected				
Great and introduces self to woman and companion	385	67.9	182	32.1
Encourages companion to remain with woman	387	68.3	180	31.7
Encourages woman and her companion to ask questions	337	59.4	230	40.6
Responds to questions with promptness, politeness & truthfulness	369	65.1	198	34.9
Explains what is being done & to expect throughout LAD	356	62.8	211	37.2
Gives periodic updates on status and progress of labor	389	68.6	178	31.4
Allows the woman to move about during labor	419	73.9	148	26.1
Allows woman to assume position of choice during birth	312	55.0	255	45.0
Obtains consent or permission prior to any procedure	350	61.7	217	38.3
3. **Confidentiality and privacy is protected**				
Confirms patient files are stored in locked cabinets with limited access	531	93.7	36	6.3
Uses curtains or other visual barrier to protect woman	518	91.4	49	8.6
Uses drapes/covering appropriate to protect woman’s privacy	487	85.9	80	14.1
4. **The woman is treated with dignity and respect.**				
Speaks politely to woman and companion	513	90.5	54	9.5
Allows woman and her companion to observe cultural practices as much as possible	449	79.2	118	20.8
Never makes insults, intimidation, threats, shouted at, scolded, laughed, scorned or coerces woman or her companion	442	78.0	125	22.0
5. **The woman receives equitable care, free of discrimination**				
Speaks to the woman in a language and at a language-level that she understands	547	96.5	20	3.5
Doesn’t show disrespect to women based on any specific attribute	519	91.5	48	8.5
6. **The woman is never left without care**				
Encourages woman to call if needed	474	83.6	93	16.4
Comes quickly when woman calls or after decision	397	70.0	170	30.0
Never leaves woman alone or unattended	353	62.3	214	37.7
7. **The woman is never detained or confined against her will**				
Facility doesn’t have a policy to detain women who don’t pay.	564	99.5	3	0.5
Don’t been forced to stay against your will	563	99.3	4	0.7

### Factors associated with Respectful Maternity care during Childbirth

The result of the bivariate analysis showed that; respondent’s educational status, religion, residence, and sex of provider are socio-demographic factors that are found to be significant with RMC at a p-value of 0.2 or less. Level of health institutions, requesting for consent, duration of stay, visit type of current delivery, number of institutional delivery, waiting time, current pregnancy status, number of attending personnel, discussion about place of delivery, availability of cultural practices, time of delivery and involvement in decision-making were other factors identified at *p*-value 0.2 or less.

On multivariable logistic regression; the type of health institutions, taking consent before procedure, current pregnancy status, number of health workers during childbirth, discussion on the place of delivery with health worker during ANC, time (shift) of delivery, duration of stay at health institutions, involvement in decision-making and waiting time to be seen by health workers were found to be significantly associated with respectful maternity care at *P*-value of ≤ 0.05.

This study identified that those women who gave birth at health centers were 5 times (AOR = 5.44, 95% CI:2.93, 10.08) more likely to receive respectful care as compared to those who gave birth at a general hospital. Women who stayed 13 to 24 h and more than 24 h at health facility were 2.1 and almost 2 times more likely to receive respectful maternity care than those who stayed 12 h or less (AOR = 2.10, 95%CI:1.24,3.56) and (AOR = 1.94, 95%CI: 1.08,3.46) respectively.

This study revealed that women whose current pregnancy was unwanted were 5.56 times more likely to get RMC than those of a wanted pregnancy (AOR = 5.56, 95%CI: 2.56–12.11). Women who had discussed on the place of delivery with health workers during ANC visits were 4.42 times more likely to receive RMC during labor and Childbirth than those who did not (AOR = 4.42, 95%CI: 2.15–9.11).

In the current study the number of attending health care providers was found to affect RMC; in which those who were attended to by 2 or fewer providers were 2.23 times more likely to receive RMC than those who were attended by 3 or more (AOR = 2.23, 95%CI: 1.30,3.82).

Taking consent before doing a procedure was found to affect RMC, which indicated that women who gave their consent before the procedure were 3.45 times more likely to get respectful care than those who did not consent (AOR = 3.45, 95%CI:1.56–7.61). Women who gave birth during the day time were 5.56 times more likely to receive respectful care than nighttime shift (AOR = 5.56, 95%CI: 3.47–8.91).

Those women who got involved in decision-making about their care were found to be 8.24 times more likely to receive respectful care than those who weren’t involved (AOR = 8.24, 95%CI: 3.63–18.67). Women who were satisfied with their current waiting time to be seen by health workers were 2 times more likely to get RMC than those who weren’t (AOR = 2.08, 95%CI: 1.02–4.25) (Table 4).

**Table 4:** Factors associated with Respectful Maternity care during labor and childbirth at health facilities in West Shewa Zone, Oromia region, central Ethiopia, 2018

Variables	Received RMC service		Adjusted OR 95% CI	Adjusted OR 95% CI	***P***-value
	Yes	No			
Type of health Facility					
Health Center	126(47.9)	137(52.1)	2.46(1.59,3.80)	5.44(2.93,10.08)	0.000*
Primary Hospital	37(23.6)	120(76.4)	0.82(0.49,1.38)	1.13(0.61,2.09)	0.686
General Hospital	40(27.2)	107(72.8)	1	1	0.000
Asked for consent before the procedure					
Not asked me	14(17.7)	65(82.3)	1	1	0.001*
Yes asked	103(42.9)	137(57.1)	3.49(1.85,6.56)	3.45(1.56,7.61)	0.002*
No procedure	86(34.7)	162(65.3)	2.46(1.30,4.64)	1.51(0.69,3.26)	0.299
Is current pregnancy wanted					
Yes wanted	173(34.1)	334(65.9)	1	1	0.000*
Not wanted	30(50.0)	30(50.0)	1.93(1.12,3.30)	5.56(2.56,12.11)	
Number of health workers during childbirth					
Two or less	132(42.2)	181(57.8)	1.88(1.31,2.67)	2.23(1.30,3.82)	
Three or more	71(28.0)	183(72.0)	1	1	0.003*
Discussed on place of delivery with health worker during ANC					
Yes	185(40.0)	277(60.0)	3.22(1.88,5.54)	4.42(2.15,9.11)	
No	18(17.1)	87(82.9)	1	1	0.000*
Time (shift) of delivery					
Day time	148(49.2)	153(50.8)	3.71(2.55,5.38)	5.56(3.47,8.91)	
Nighttime	55(20.7)	211(79.3)	1	1	0.000*
Duration of stay at health facilities					
12 h or less	79(28.5)	198(71.5)	1	1	0.009*
13 to 24 h	68(46.3)	79(53.7)	2.15(1.42,3.27)	2.10(1.24,3.56)	0.005
More than 24 h	56(39.2)	87(60.8)	1.61(1.05,2.46)	1.94(1.08,3.46)	0.025
Involvement in decision-making					
Involved	194(40.6)	284(59.4)	6.07(2.97,12.38)	8.24(3.63,18.67)	
Not involved	9(10.1)	80(89.9)	1	1	0.000*
Waiting time to be seen by the health worker`					
Yes satisfied	188(38.0)	307(62.0)	2.32(1.28,4.22)	2.08(1.02,4.25)	
Not satisfied	15(20.8)	57(79.2)	1	1	0.045*

*variables found to be statistically significant at a *p*-value of less than 0.05

## Discussion

The current study indicated that overall, only one-third of women received RMC during labor and childbirth at public health institutions in the West Shewa Zone [35.8% (95% CI: 31.7–39.7)]. This is in line with the study done in Bahirdar town 32.9% of mothers experienced respectful and non-abusive care [25]. However, this is higher than the study done in Addis Ababa where 21.4% of respondents received respectful and non-abusive care [18]. This variation might be due to the difference in the study setting and study population in which Addis Ababa’s study was only limited to the town and they excluded those mothers who had an elective or emergency cesarean section.

The current finding is lower than the study done in Bahirdar town, Ethiopia in which 57% of women experienced RMC [25]. In Northern Ethiopia, 22% reported at least one incident of DRA [30]. In Addis Ababa 82.4% had received RMC [31], and in another study in Addis Ababa at least one form of DRA in 36% of the observations [28]. Health centers in two regions of Ethiopia also showed 21.1% of respondents reported DRA [32] and a systematic review in Ethiopia also indicated 49.4% of DRA [21]. The current finding (35.8%) is also lower than the study done in urban Tanzania (15%) of postpartum women reported at least one instance of DRA [17]. Another study done in Tanzania 19.48% reported DRA on exit from the health facilities and 28.21% on follow-up interview in the community [12]. From the study done in Kenya, 20% of women had reported any form of DRA [33]. In another multi-level study in Kenya DRA decreased from 20 to 13% [34]. The possible reason for this variation might be due to the difference in the study setting in which the current study was conducted both in urban and rural settings unlike studies done in Bahirdar town, Addis Ababa city and Tanzania. The presence of projects working in the area in Tanzania, Kenya, northern Ethiopia, a study by Kathleen P et al. Additionally, the variations in the health care system with Tanzania and Kenya might be the possible reasons.

Although RMC is a universal right of every childbearing woman in the health care system, they may still experience disrespect and abusive care during childbirth [3, 4]. In the current study more than four-fifths of the women, received equitable care and their confidentiality and privacy protected. Three fourth of women were protected from physical harm or ill-treatment. Women never left without care and the right to consented care were the least respected categories of RMC.

A study done in Bahirdar indicated that; providing a discrimination-free, friendly and abuse-free care to be the commonly practiced category of RMC [25]. Another study identified physical abuse and non-consented care were the commonly experienced categories of DRA [23]. In Addis Ababa study showed that in one-third of the women physical harm or ill-treatment were not protected, while women left without care constitute almost two-fifth. In 33% of hospitals and 9.4% of health centers their privacy was not protected and 94.8% of women have experienced non-consented care [18]. In another study by Kitaw M. et al., one in nine women received discrimination-free and with no abandonment of care. But, only one-fifth of women received dignified care during their childbirth [31]. The study in two regions in Ethiopia indicated non-consented care, lack of privacy, non-confidential care, were the commonly observed forms of DRA [32]. The pooled data in Ethiopia, also indicated, abandonment of care, non-confidential care and physical abuse as common forms of mistreatments [21]. These shows the proportion of each category of RMC among different studies in Ethiopia varies.

These variations might be due to the differences in the study setting, resulting in the different prevalence of RMC categories among health institutions (Hospitals vs. Health centers), town versus rural settings and different cadres in the health care system. This is highlighting as there is a need for specific local interventions for different settings to lower the forms of DRA in Ethiopia.

From a study done in Kenya and Tanzania relatively lower proportion of all forms of DRAs were reported, indicating that a large proportion of women in Ethiopia are experiencing disrespectful and abusive care during labor and childbirth [17, 33].

This study identified that the type of health facility in the health care system was significantly associated with RMC. Women who gave birth at health centers were more likely to receive respectful care as compared to those who gave birth in the general hospital. This is in-line with the study done in Addis Ababa that indicated as there was a significant difference between the health centers and hospitals [18] and from the study done in Malawi the odds of a health provider shouting at a woman were lower in health centers compared to hospitals [35] This is may be due to the presence of more number of caseloads compared to the available number of human resources at hospitals than in health centers.

Maternal stay at the health facility was also found to be significantly associated with RMC during labor and childbirth. Mothers who stayed 13 to 24 h and more than 24 h at health institutions were almost 2 times more likely to receive RMC than those who stayed less than 12 h. This finding is in agreement with a study done in Tanzania where women who stayed less than 1 day in the facility for delivery were 1.35 times more likely to report experiences of DRA [12]. The reason for this might be due to the fact that those women who stayed longer may become familiar with the health workers and are more likely to receive customary services.

Women who gave birth during the day time were 5.56 times more likely to receive respectful care than a nighttime shift. In agreement with this, a study done in Kenya identified delivering at night was associated with a higher risk of DRA and had greater odds of reporting physical abuse than those delivering during the day [34]. This might be due to the fact that during day time there are more resources/infrastructures available and the number of health workers than at nighttime in which only one health worker might be assigned for duty in health centers and also very weak supervision from senior health workers and managers during nighttime.

This study revealed that women whose current pregnancy was unwanted were 5.56 times more likely to get RMC than those of a wanted pregnancy. In agreement with this study conducted in Bahirdar also showed the odds of experiencing DRA was 76% less among those unplanned or unwanted pregnancy [25]. This is may be due to the fact that those women with an unwanted pregnancy were less likely to be worried about the outcomes and were multiparous in the current study.

The result also has shown that women who had discussed on a place of delivery with health workers during ANC were 4.42 times more likely to receive RMC than those who did not. A study done in Bahirdar indicated that respondents with fewer than 4 ANC visits were more likely to have been disrespected and abused than those with ≥ 4 ANC visits [23]. The reason for this might be because women who had ANC and discussed on a place of delivery were more likely to be familiar with the health care providers, since majority of them in the current study gave birth at the same facility.

In the current study, the number of attending health care provider was found to be significantly associated with RMC during labor and childbirth. Mothers who were attended to by 2 or fewer providers were 2.23 times more likely to receive RMC than those who were attended by 3 or more. This is may be due to mothers do not want to show their private body to more number of providers.

Taking consent before doing a procedure was found to affect RMC. It was shown that women who gave their consent before the procedure were 3.45 times more likely to get respectful care than those who did not consent. Those women who got involved in the decision-making about their care were found to be 8.24 times more likely to receive respectful care than those who were not involved. The reason might be that in our study area there is a wrong perception among some health care providers that mothers might feel more pain if informed before performing procedures like episiotomy, manual removal of placentas and others. Thus, they prefer doing those procedures without informing the clients and even sometimes performs without providing analgesia. This result also highlights the need for involving mothers in all types of care they receive from health facilities.

Women who were satisfied with their waiting time to be seen by health worker were 2 times more likely to get RMC than those who were not. The reason for this might be due to women who were not satisfied were likely to feel as they were neglected or left without care if not seen by health workers after admission.

Limitation of the study: Though the problem of recall bias was minimized by conducting exit interview for postpartum mothers immediately; the current study is not free of social desirability bias in which some mothers may report the service as positive experiences while they are in the health facilities. As a strength, the study tried to cover a large number of health facilities including health centers and hospitals in the West Shewa zone providing services for over 2.3 million peoples.

## Conclusions

The proportion of RMC during labor and childbirth in health institutions in the west Shewa zone was low. Giving birth at the health center, discussion on the place of delivery with health workers during ANC, day time delivery, longer duration of stay at health facility (≥ 13 h), involvement in decision-making, consent before the procedure, current pregnancy not wanted, presence of < 3 health workers during childbirth, and satisfied with waiting time to be seen by a health worker were found to be significantly associated with RMC.

Therefore, health institutions and all other stakeholders should give due emphasis on creating awareness of care providers on the standards and categories of RMC, and emphatically consider those identified factors for intervention. Additionally, monitoring and reinforcing accountability mechanisms for health workers to avoid mistreatments, and supporting them to provide the service with respect and compassion during labor and childbirth. Further research involving observation is also recommended to get more information about RMC services.

## Data Availability

Datasets used in the current study are available from the corresponding author upon reasonable request.

## Abbreviations

ANC: Ante Natal Care
AOR: Adjusted Odds Ratio
CRC: Caring, Respectful and Compassionate
DRA: Disrespect and Abuse
HSTP: Health Sector Transformation Plan
LAD: Labor And Delivery
RMC: Respectful Maternity Care
